# Pre‐industrial Use of Bauxite by Late Gothic Goldsmith Masters: Analytical Evidence and Experimental Study

**DOI:** 10.1002/cplu.202500044

**Published:** 2025-03-25

**Authors:** David Hradil, Janka Hradilová, Petr Bezdička, Ivan Razum

**Affiliations:** ^1^ ALMA Laboratory Institute of Inorganic Chemistry of the Czech Academy of Sciences Husinec-Řež 1001 250 68 Řež Czech Republic; ^2^ Croatian Natural History Museum in Zagreb Demetrova 1 10 000 Zagreb Croatia

**Keywords:** X-ray powder micro-diffraction, gilding, bauxite, diaspore, Late Gothic art

## Abstract

The turn of the 15^th^ and the 16^th^ century was marked by turmoil including invasion of the Turks in the Balkans. Consequently, trade routes to the Mediterranean were disrupted, resulting in sudden demand for alternative sources of imported materials. For example, direct import of potassium alum had been gradually replaced by its local production from alunite, alum schists or grey pyritic bauxite. It has now been proven that concurrently extracted red bauxite had been used as a substitute for imported high‐quality red clay, the so‐called Armenian bole, employed by Central European painting workshops in the preparations for gilding (“poliments”). Importantly, connection with the documented mining of grey bauxite in Croatian Minjera was evidenced by unique finding of diaspore together with dominant boehmite. Mineralogical analyses were performed by X‐ray powder micro‐diffraction and reference bauxites from Istria and Balkan Peninsula were used to evaluate their technological suitability for gilding. As it was found that the earliest appearance of boehmite in poliment dates back to 1470, the beginning of bauxite mining in Europe is shifted to the period shortly after the fall of Constantinople in 1453, and it also represents the oldest known evidence of the use of bauxite raw material in technology.

## Graphical abstract

First analytical evidence and replication of yet undescribed substantial change in gilding technology in the early modern period ‐ the replacement of imported red clay (bole) with locally available bauxite. Proof of its source ‐ Croatian Minjera, according to a unique find of mineral diaspore. Earliest documented use of bauxite in technology ever, important for dating anonymous paintings.

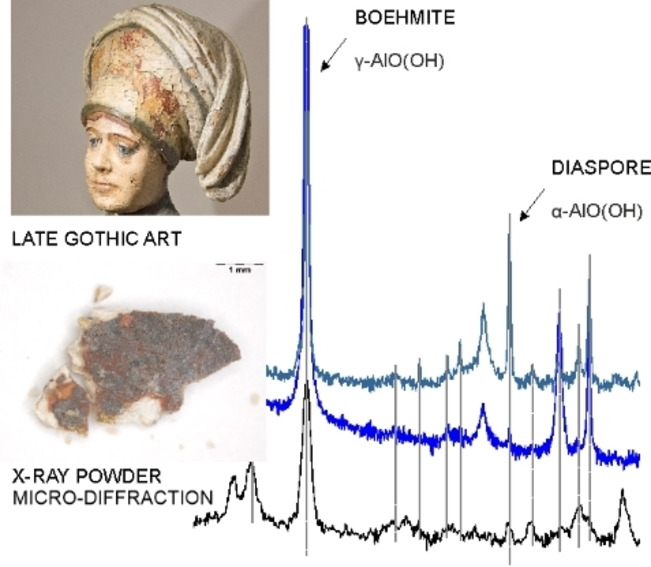

## Introduction

In fine arts, aluminium‐rich materials encompass Al (oxo)hydroxides and/or sulphates, either alone or mixed with alumosilicates (earths and clays). However, Al‐rich materials in historical painting cannot be perceived as one coherent group. The most well‐known and most widely used was potassium alum (KAl(SO_4_)_2_ ⋅ 12H_2_O), employed for precipitation of organic lakes. The final product contains the dye, aluminium hydroxide and residual alum. Other examples, not related to organic dyes, are much less represented. For example, volcanogenic weathered and sedimentary rocks in Italy may contain minerals of the jarosite‐alunite series, which may appear as an admixture in natural ferruginous reds and ochres. Whether the admixture of alunite (KAl_3_[(OH)_6_|(SO_4_)_2_]) and/or jarosite (KFe_3_[(OH)_6_|(SO_4_)_2_]) in a painting layer was intentional or not, is difficult to decide. These sulphates were identified in some Italian and Spanish artworks, e. g., alunite was found as an admixture in ground layer of a Venetian Baroque “veduta” (i. e. realistic view of a city).^[1]^ Intentional use can be indicated by higher proportion of the sulphate, which has been evidenced already at the beginning of the 17^th^ century. A substantial amount of alunite, together with hematite (Fe_2_O_3_) and gypsum (CaSO_4_.2H_2_O), was found in the ground layer of Caravaggio's painting *The Beheading of St. John the Baptist*, painted in 1608 in Malta (although in his previous work, northern Italian pottery clays were used for that purpose). Alunite is associated with the Malta region also in the paintings by Mattia Preti, however, only as an admixture in the local *Globigerina* limestones material.[^2^, ^3^]

Although alum has already been mined by ancient Egyptians, it is only rarely found in its natural form and its production from other raw materials prevailed, either from alunite or, later on, from alum schists. After 1453 (the fall of Constantinople), major problems arose regarding future supplies of alum to Europe from the Mediterranean to meet the ever‐increasing demand. The Levant was under the control of Turks and Western Europe was faced with the need to find its own alternative resources. As a result, the largest alunite deposit in Europe – Tolfa (north of Rome) – has been discovered in 1460–61^[4]^ and, in the similar period of time, beginning of alum mining in the Zaragoza area, Spain, has been documented.^[5]^ Besides alunite, also pyrite‐rich schists became a raw material for the production of alum in the 16^th^ century – mined in many places in Europe. In Czechia, for example, according to local chronicles, mining began near Chomutov in 1558 and in Hromnice near Pilsen in 1578.

One can only theorize whether bauxite, from which alum is produced today, could have been a source material also in the past. There is no relevant information in the literature, with only one exception. Marušić *et al*.^[6]^ were the first to publish that in the valley of the river Mirna in Istria, Croatia, beneath the Castle of Sovinjak near Buzet, grey pyritic bauxite has been exploited underground already in the 16^th^ century to make alum and vitriol (sulphuric acid). Thanks to this, Minjera is considered to be the first bauxite mine in the world.[^7^, ^8^]

It has already been described^[9]^ that the red preparations for gilding (“poliments”) of early 16^th^ century artworks from the regions of German‐speaking communities in Central Europe (Bohemian‐Saxon border, Spiš in Slovakia, Transylvania in Romania) are usually thicker than traditional mediaeval ones and contain typical bauxite minerals gibbsite – Al(OH)_3_ and/or boehmite ‐ γ‐AlO(OH). Could this indicate the use of newly available bauxite, which was found to be a satisfactory substitute for expensive Armenian bole (red clay) imported from the Mediterranean? Can we imagine that while the grey bauxite was used to make alum, the surrounding red one was sold to goldsmiths and painters as a by‐product? As these two types of bauxite had to be mined together due to their close genetic association, it is a viable idea. Grey bauxite was formed from red bauxite by diagenetic transformation only in some parts of the deposit with reducing environment in the presence of organic matter, with red hematite being transformed into pyrite and marcasite (both FeS_2_).^[8]^

The exact year of beginning of the mining in Minjera is not known. Researchers still rely only on one literary source, a short communication of bishop Tommasini from Cittanova (today Novigrad) written in 1646, who states that “*about eighty years ago*, *the German miners abandoned their alum mine near SovignaccolSovinjak*”. This means that the first stage of mining had taken place already before ca. 1566. Regardless, there is one more seemingly minor circumstance. As mentioned by Marušić *et al*.,^[6]^ prospecting adits in Minjera were dug with knowledge of geology to cross the bauxite bodies, which were hidden, covered by younger Eocene sediments and not visible at any outcrop. Therefore, the mine opening was not accidental, but highly experienced. It means that the Saxon miners, who at that time were fleeing the Turks and migrating from the Balkan Peninsula to the mediaeval Republic of Venice (of which Istria was a part of), knew what they were looking for and why. In the Balkans, where there are many other occurrences of bauxite, Saxon miners have been active since the 12^th^ century. This would mean that Minjera was not necessarily the very first bauxite mine exploited.

In order to demonstrate and verify the possibility of using red bauxite for painting and gilding purposes as early as the beginning of the 16^th^ century, phase composition of bauxites from Istrian and Dinaric karst was compared with the composition of poliments under gilding created in rich mining regions in Central Europe in the period from 1470 to 1550. All the mentioned regions in Istria, the Balkans and Central Europe were at that time already linked by the activity of Saxon prospectors and miners, including numerous trade relations. In order to correlate materials of poliments with their possible sources, a compositional data approach was used.^[10]^ This comparative study also included technological tests imitating historical gilding procedures.

## Results and Discussion

### Collection of Samples and Micro‐Samples

**Figure 1:**
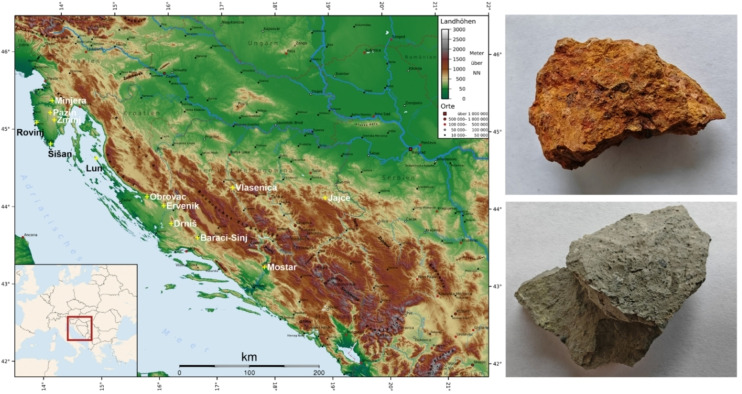
Map of bauxite sampling sites in the Istrian and Dinaric Karst; map from https://en.wikipedia.org/wiki/Dinaric_Alps, supplemented with names of sampling locations; rock samples of red‐orange (MIN 4–1) and grey (MIN 1–1) bauxite from Minjera on the right

Based on the assumption that bauxite was extracted by Saxon miners in the region of Istria and, eventually, also the Balkan Peninsula already in the 16^th^ century, rock samples including economically important bauxite accumulations in forelands of the Dinaric Alps (Istrian and Dinaric karst) were selected for comparative analysis. Deposits located in present‐day Croatia (including historical Minjera) and Bosnia and Herzegovina (Mostar, Vlasenica and Jajce) were included. They were provided by the Croatian Natural History Museum (CNHM) and the University in Zagreb, Faculty of Mining, Geology and Petroleum Engineering (RGN), and some of them were personally collected in 2006 and 2024. Their list is given in Table 1 and the locations are depicted in Figure 1.

**Table 1:** Description of reference samples of red and grey bauxites selected for comparative research.

Code/colour	Deposit/geological age/provider^[a]^
Istrian karst[^11^, ^12^]	
HR‐14G/grey	Rovinj/Jurassic/personally collected
HR‐14R/red	Rovinj/Jurassic/RGN
HR‐15G/grey	Minjera/Lower Palaeogene/ RGN
HR‐15R/red	
MIN 1–1/grey	Minjera/Lower Palaeogene/personally collected
MIN 4–1/orange	
HR‐4/red	Pazin/Lower Palaeogene/CNHM Inv.no. 600:ZAG; 6751:MP1
HR‐5/red	Žminj/Lower Palaeogene/CNHM Inv.no. 600:ZAG; 6469:MP1
HR‐3/red	Šišan/Lower Palaeogene/ CNHM Inv.no. 600:ZAG; 6467:MP1

Dinaridic high karst – North Dalmatian Islands/Basin[^11^, ^12^, ^13^, ^14^, ^15^]	
HR‐6/red	Lun (Pag)/Lower Paleogene/CNHM Inv.no. 600:ZAG;10901:MP1
HR‐1/red	Ervenik/Lower Palaeogene/ CNHM Inv.no. 600:ZAG; 6470:MP1
HR‐2,8/red	Drniš/Lower Palaeogene/ CNHM Inv.no. 600:ZAG; 6472:MP1 Inv.no. 600:ZAG; 4360:MP1
HR‐9/red	Obrovac/Lower Palaeogene/ CNHM Inv.no. 600:ZAG;10903:MP1
HR‐7/red	Baraći‐Sinj/Lower Palaeogene/CNHM Inv.no. 600:ZAG;10905:MP1
HR‐12/red	Mostar (BiH)/Lower Palaeogene/CNHM Inv.no. 600:ZAG; 6948:MP1

Inner karst[^11^, ^16^]	
HR‐11/red	Jajce (BiH)/Lower‐Upper Cretaceous/CNHM Inv.no. 600:ZAG; 6747:MP1
HR‐13/red	Vlasenica (BiH)/Lower Cretaceous/CNHM Inv.no. 600:ZAG;10902:MP1

[a] all deposits are located in Croatia, except those marked BiH (= Bosnia and Herzegovina); RGN=University in Zagreb, Faculty of Mining, Geology and Petroleum Engineering, CNHM=Croatian Natural History Museum)

One part of each reference bauxite sample was embedded in epoxy resin, ground, and polished to obtain cross‐section suitable for microscopic observations and analysis; part of the remaining material was gently powdered in an agate mortar and subjected to phase analysis. For the purposes of technological experiments, red clay from Hořenec near Chomutov, Czech Republic (H111, the so‐called Czech bole, personally collected) and Sardinian smectite‐kaolinite red clay with commercial name “Rosso Sartorius” (40490, Kremer Pigmente, Ltd., Germany) were used together with selected bauxites. Their mineralogical composition was already reported.^[17]^ Gypsum (CaSO_4_.2H_2_O) personally collected from the historical mining area near Bologna), animal glue (Kremer Pigmente, Ltd., Germany), gold foil (Krustashop, s.r.o. Czech Republic) and oak plates of 10x5x1 cm size were also used in technological tests. This study also encompasses micro‐fragments of gilding/silvering from polychrome sculptures and panel paintings created at the turn of the 15^th^ and 16^th^ centuries (between 1470 to 1550). They represent the art production of two regions closely connected with the activities of Saxon prospectors and miners ‐ Spiš region in Slovakia (Zips in German, formerly Upper Hungary), the area of western Bohemia and the Ore Mountains at the Czech‐Saxon border and the area of eastern Silesia (on the recent Czech‐Polish border). The studied artworks and samples are listed in Table 2.

**Table 2:** Description of painted artworks and studied micro‐samples of gilding/silvering on red poliment.

Code	Artwork, place/attribution	Date of creation	Location of samples/type of metal
J2056	*Virgin Mary* (polychrome wooden sculpture), Roman Catholic Church of St. George, Spišská Sobota, Spiš region, SK/signed „iohannes“	around 1470	2: brocade on the Virgin Mary's robe/gold
J1810	*Death of the Virgin Mary (*polychrome group of sculptures + panel paintings), Roman Catholic Church of St. Bishop Martin, Spišské Podhradie, Spiš region, SK/unknown origin	around 1478	5: (sculpture) right shoulder of the Virgin Mary/gold 17: (sculpture) red cloak of an apostle/silver + red glaze 21: (panel painting) St. Ladislaus – gilded background/gold 22: (panel painting) St. John the Almsman – gilded background/gold
J2439	*Adoration of the Magi* (polychrome group of sculptures), Roman Catholic Church of St. Bishop Martin, Spišské Podhradie, Spiš region, SK/unknown origin	1497	1: The Kneeling King Kaspar/gold
J1324^[a]^	Triptych “*Death of the Virgin Mary*” and predella (panel painting), Royal chapel, Italian Court at Kutná Hora/Kuttenberg, CZ/South‐German workshop (probably Nuremberg School)	1497	9: inner frame/gold
J1328^[a]^	*Mourning of Christ*, according the engraving by Albrecht Dürer (wooden relief), Minorite Monastery at Brno, CZ/South‐German workshop (probably Ulm)	1500–1510	13: inner side of the drapery Virgin Mary's drapery/gold
J2055	*St. Antoninus* (polychrome wooden sculpture), Roman Catholic Church of St. George, Spišská Sobota, Spiš region, SK/Monogramist H.T.	1503	3: the robe of St. Antoninus/silver 5: back side of the sculpture/poliment without metal
J1954	*St. George Altar* (polychrome wooden sculptures + panel paintings), Church of St. James, Levoča, Spiš region, SK/workshop of Master Paul of Levoča	1516	8: (panel painting) St. Christopher – gilded background/part gold (*Zwischgold*) 17: (sculpture) Last supper – silver plate with lamb/silver with glaze
J1605^[a]^	Crucified Christ (polychrome wooden sculpture), Spišské Vlachy, SK/Master Paul from Levoča	around 1520	4: Christ's loincloth (new sampling)/gold
J1606^[a]^	*St. John Chrysostom –* niche behind the sculpture (painting), St. Johns’ Altar ‐ St. James Church in Levoča, Spiš region, SK/Hans T. ‐ workshop of Master Paul from Levoča	around 1520	6: background behind the sculpture/poliment without metal
J1607^[a]^	*St. John the Baptist* (polychrome wooden sculpture), St. Johns’ Altar ‐ St. James Church in Levoča, Spiš region, SK/Master Paul from Levoča	around 1520	2: back side of the sculpture/poliment without metal
J2012	*Beheading of St. Catherine* ‐ central panel of the Triptych of St. Catherine of Alexandria (panel painting), Regional Museum and Gallery in Most, CZ/Master of Dippoldiswalde	1520	7: gilded background/gold
J1847	*St. Barbara* (polychrome wooden sculpture + architecture), St. Barbara Altar, Regional Museum in Chomutov, CZ/workshop of Hans Hesse (newly attributed)	1524–1525	5: gilded frame of the niche/silver
J2040	*Angel* (polychrome wooden carving), St. Ann's Altar, Church of St. James, Levoča, Spiš region, SK/workshop of Master Paul of Levoča	1525	1: ornament/silver 2: hem of the costume/silver with red glaze
J0914^[a]^	*Nativity (Birth of Christ)* (panel painting), St. Martin's Altar, Church of St. Bishop Martin of Tours, Lipany, Spiš region, SK/signed H.E.R., workshop of Master Paul from Levoča	1526	10: gilded background/gold
J1841	*Tondo with St. Ann of Sorrows* (polychrome wooden relief), Regional Museum and Gallery in Most, CZ/unknown	before 1530	1, 3: red pillow/silver with red glaze
J1843	*Votive panel with Assumpta* (panel painting), Regional Museum and Gallery in Most, CZ/Master IW	1538	6, 7: original frame of the painting/silver with yellow glaze (*Waschgold*)
J1963	*St. Ann teaching the Virgin Mary* (polychrome wooden sculpture), Silesian Museum in Opava, CZ/unknown German master	1525–1550	4b: white cap (original gilding overpainted)/silver with red glaze

[a] artworks previously investigated by Hradil *et al*.^[9]^

**Figure 2:**
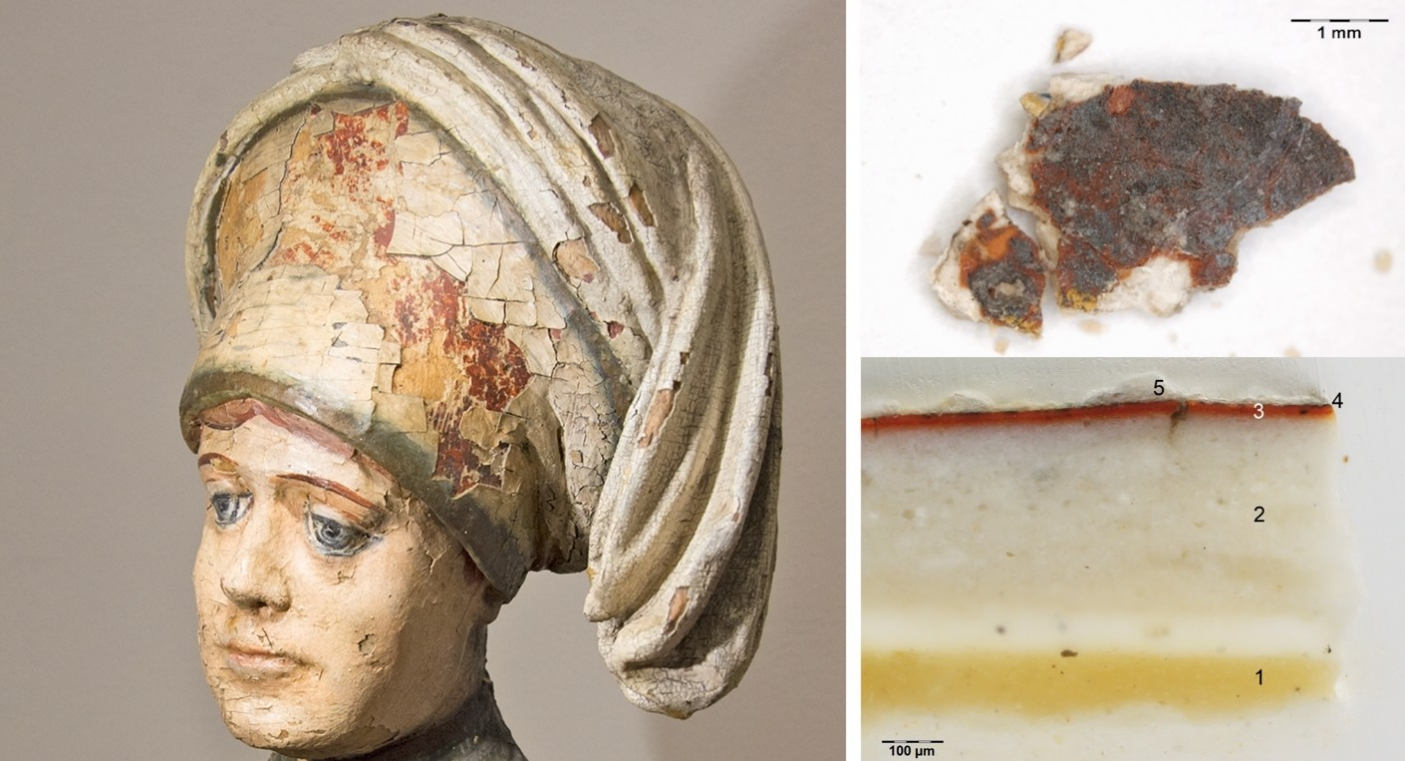
Head of St. Ann from the sculpture *St. Ann is teaching the Virgin Mary* (J1963) with clearly visible remains of silvering on red‐orange poliment emerging from beneath the fading white overpainting (left) and one micro‐fragment of this silvering in top view – used for phase analysis (top right) and in its cross‐section with layer stratigraphy uncovered – used for elemental analysis (bottom right); 1 – glue insulation, 2 – chalk ground, 3 –poliment, 4 – corroded silver foil with remains of a red glaze, 5 – wax (conservation) (photos J. Hradilová)

The selected collection includes works by important Late‐Gothic artists active in Central Europe, such as the painter Master IW, a pupil of Lucas Cranach the Elder,^[18]^ or the sculptor and carver Master Paul of Levoča, an alleged pupil of Veit Stoss^[19]^ All the micro‐fragments of gilding were split into two parts – one part was embedded in synthetic polyester resin and prepared in the form of cross‐section for the purpose microscopic observations and analysis and the second remaining part was not treated and directly subjected to the phase micro‐analysis. As an example, the polychrome sculpture *St. Ann teaching the Virgin Mary* from 1525–1550 is presented in Figure 2, where the micro‐fragment and the layer stratigraphy in its cross‐section are also depicted.

### Analysis of Expandable Clay Structures

Results of X‐ray powder diffraction (XRPD) and/or micro‐diffraction (μXRPD) analysis of reference bauxite samples and artwork micro‐samples are summarized in Tables 3 and 4. In addition to Al and Fe oxohydroxides (hematite – Fe_2_O_3_, goethite – FeO(OH), gibbsite – Al(OH)_3_, boehmite ‐ γ‐AlO(OH), dispore ‐ α‐AlO(OH) as well as Ti oxides (anatase – TiO_2_), clay minerals, especially kaolinite – Al_2_Si_2_O_5_(OH)_4_, are also typical components of red bauxites. Furthermore, namely bauxites from Rovinj are characterized by increased proportions of Mg‐chlorite ‐ Mg_5_Al(AlSi_3_O_10_)(OH), and in the literature variable presence of illite ‐ K_0.6‐0.85_(Al,Mg)_2_(Si,Al)_4_O_10_(OH)_2_, vermiculite ‐ (Mg,Fe,Al)_3_(Al,Si)_4_O_10_(OH)_2_ ⋅ 4(H_2_O) and mixed‐layered structures containing chlorite, vermiculite and illite were also reported^[20]^

**Table 3:** Results of XRPD analysis of reference bauxites – estimated contents of mineral phases in wt.%.

Sample code and location	Gibbsite Al(OH)_3_	Boehmite γ‐Al O(OH)	Diaspore α‐Al O(OH)	Hematite Fe_2_O_3_	Goethite α‐Fe O(OH)	Pyrite FeS_2_	Anatase TiO_2_	Rutile TiO_2_	Kaolinite Al_2_Si_2_O_5_(OH)_4_	Clinochlore Mg_5_Al(AlSi_3_O_10_)(OH)_8_	Gypsum CaSO_4_.2H_2_O	Calcite CaCO_3_	Natroalunite NaAl_3_(SO_4_)_2_(OH)_6_	Jarosite KFe_3_(SO_4_)_2_(OH)_6_	Szomolnokite FeSO_4_ ⋅ H_2_O	Melanterite FeSO_4_⋅7(H_2_O)
HR‐14G Rovinj (grey)		42.4					2.3	0.7	45.4	5.9	0.5			2.1		
HR‐14R Rovinj		50.2		11.9			1.8	2.0	28.2	6.0						
HR‐15G Minjera (grey)	2.1	71.7	1.8			8.5	2.8	0.9	8.4						3.6	0.3
HR‐15R Minjera	+	74.1	0.7	18.4	2.1		2.1	0.6	2.1							
MIN 1–1 – A Minjera (grey)		9.1	15.7			7.9	2.5	1.1	60.1	3.4						
MIN 1–1 – B Minjera (grey)		9.4	15.6			9.1	2.6	0.9	59.9	2.3						
MIN 4–1 – A Minjera		42.8	20.9	6.7	10.7		3.6	1.8	13.5							
MIN 4–1 – B Minjera		46.4	17.8	7.1	9.5		3.5	2.3	13.6							
HR‐4 Pazin		65.7					4.9	1.2	18.3				9.9			
HR‐5 Žminj		75.8		20.6			2.7	0.9								
HR‐3 Šišan		66.3		17.3	7.1		3.2	1.0	5.1							
HR‐6 Lun		49.7		21.4			2.5	0.9	25.3	0.2						
HR‐1 – A Ervenik	27.8	28.3		18.9	16.3		2.8	2.5	3.5	0.0						
HR‐1 – B Ervenik	36.2	35.4		1.4	15.1		3.5	2.5	5.4	0.5						
HR‐2 Drniš	0.8	74.2		5.8	5.8		3.8	1.3		0.4		8.0				
HR‐8 Drniš	37.3	32.0		6.2	19.7		3.2	1.8								
HR‐9 Obrovac	56.2	10.2		8.8	14.2		2.6		8.0	0.0						
HR‐7 Baraći‐Sinj	50.5	3.9		15.6	12.3		1.4		13.8	2.6						
HR‐12 Mostar	1.5	67.2		11.2	16.5		3.1	0.6								
HR‐11 Jajce		68.6		22.6			2.5	1.4	4.8			0.1				
HR‐13 Vlasenica		20.7		10.4	4.3		2.0	1.9	59.5	1.1						

^+^ identified but not quantified (sub‐limit amount) A,B in the sample code ‐ different parts of apparently heterogeneous sample Grey scale: above 20 wt.% (dark grey), 10–20 wt.% (light grey), bellow 10 wt.% (white)

**Table 4:** Results of μXRPD analysis of preparatory layers for gilding (poliments) measured on untreated micro‐fragments from the top (Figure 2) – estimated contents of mineral phases in wt.%.

Sample code	Gibbsite Al(OH)_3_	Boehmite γ‐Al O(OH)	Diaspore α‐Al O(OH)	Hematite Fe_2_O_3_	Goethite α‐Fe O(OH)	Pyrite FeS_2_	Anatase TiO_2_	Rutile TiO_2_	Quartz SiO_2_	Kaolinite Al_2_Si_2_O_5_(OH)_4_	Clinochlore Mg_5_Al(AlSi_3_O_10_)(OH)_8_	Mica, e. g. illite K_0.6‐0.85_(Al,Mg)_2_(Si,Al)_4_O_10_(OH)_2_	Expandable clay minerals (ECM) ^[a]^	Gypsum CaSO_4_.2H_2_O	Dolomite CaMg (CO_3_)_2_	Plagioclase (Na,Ca)AlSi_3_O_8_
J2056‐2		4.6		3.1					23.2	29.9		17.5		21.6		
J1810‐5		3.7		11.2	2.1		+		+	54.0		13.8	3.0	5.7	6.5	
J1810‐17		8.4	+	7.0	0.9		1.4		15.4	32.1		24.5		2.5	7.9	
J1810‐21		10.5	3.5	7.0	1.2		+		22.2	29.9		15.0		10.8	+	
J1810‐22							4.5		22.2	38.7		25.5	9.1	+		
J2439‐1				42.1			0.0	2.5	24.0	9.7		11.6		10.0		
J1324‐9 ^[b]^	7.8	15.3		8.6			11.2		6.0	30.4	7.8	5.2		7.8		
J2055‐3		1.1		3.0			+		29.7	32.7		23.8	5.0	4.7		
J2055‐5				14.3	6.8		+		16.3	38.3		11.6		12.7		
J1328‐25^[b]^	21.8	1.1		3.1			3.1		11.0	22.7	18.9	7.6		10.7		
J1954‐8	28.1	3.0		6.7	+	0.5	0.3		6.4	34.6	5.9	8.4		6.1		
J1954‐17	26.5	3.9		7.5	+		0.3		14.0	27.6	14.1	2.6		3.6		
J1605‐4^[c]^	3.7	13.6	5.2	5.8			1.0		5.8	21.7	+	21.4	9.4	5.5		7.0
J1606‐6^[b]^	17.8	4.5		8.4			7.2		18.8	19.5	6.7	10.3		6.7		
J1607‐2^[b]^	14.3	7.1		9.4			5.2		4.9	35.3	7.1	8.9		7.8		
J2012‐7		19.6		11.3			4.5		20.8	26.6		8.3		8.9		
J1847‐5	28.4			12.7			0.0		28.7	23.0		2.1		5.1		
J2040‐2		44.7		14.0			0.0		+	21.3		8.1		11.9		
J2040‐1		47.6		7.6			0.0		13.9	19.0	+	4.8		7.1		
J0914‐10^[b]^	8.7	4.5		4.3			8.0		6.2	20.8	3.7	11.8		20.3		11.7
J1841‐1				22.5			0.0		27.3	43.0		7.2				
J1841‐3				12.0			5.5		12.7	53.2		16.5				
J1963‐4b	29.6	12.0		4.2			1.4		7.8	29.2	5.2	3.0	1.7	5.9		
J1843‐6				10.6			+		9.6	72.1		7.8				
J1843‐7				6.9			0.0		12.1	69.4		3.9	7.7			

[a] ECM include smectite and interstratified structures (mixed crystals) with smectite [b] calculated from published data ^[9]^ [c] new sampling on the previously studied sculpture ^[9]^ + identified but not quantified (sub‐limit amount) Grey scale: above 20 wt.% (dark grey), 10–20 wt.% (light grey), bellow 10 wt.% (white)

A detailed analysis of clay minerals in bauxites, focusing in particular on expandable minerals (ECM, e. g. smectite group minerals or vermiculite) and on various mixed crystals with variable content of expandable components, could be very interesting from a provenance perspective. However, in our case we encounter several limits ‐ (i) no ECMs were detected in reference bauxites and (ii) ECMs detected in paint layers are already affected by interaction with the proteinaceous component of the binder, as already reported.^[21]^ It is not clear whether the resulting organo‐clay complex will react in the same way as pure clay in ethylene glycol (EG) vapour, which is the reaction widely used to distinguish expandable and non‐expandable structures (e. g. smectites from chlorites). EG enters the interlayers of expandable clay structures and increases the interplanar distance, which is manifested by a characteristic shift of basal (001) diffraction lines towards lower angles.^[22]^

Although no ECM were detected in the series of measured reference samples, they have been previously identified in some Croatian localities, e. g., Ervenik,^[13]^ or the above‐mentioned Rovinj.^[20]^ Their nature is diverse and they always occur as minor phases.

**Figure 3:**
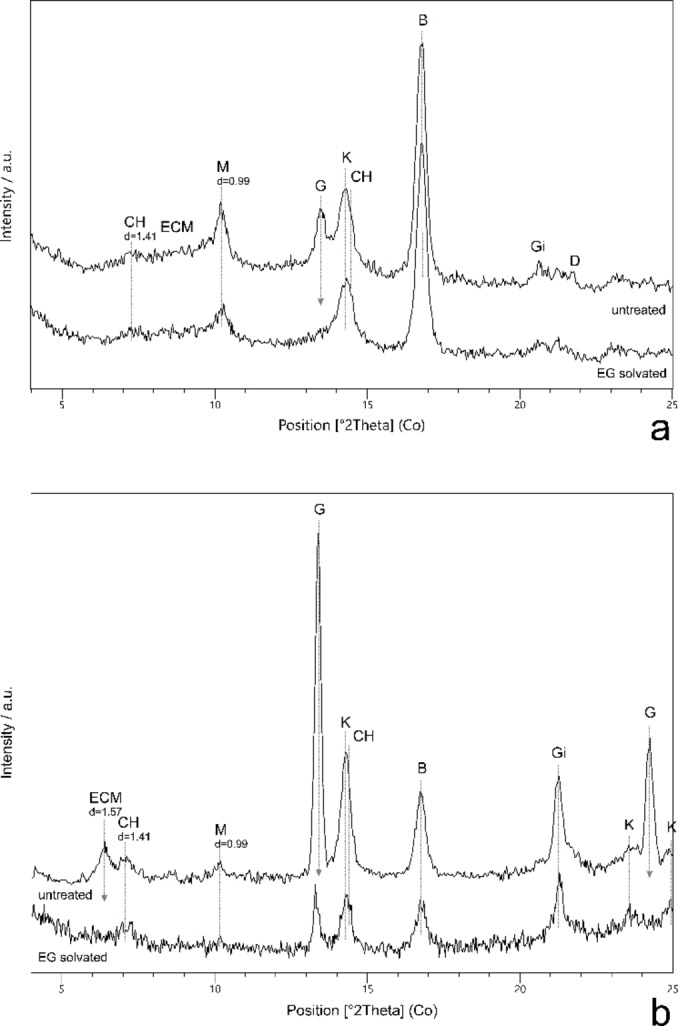
Parts of the diffraction patterns of micro‐fragments taken from polychrome statues of (a) *Crucified Christ* from Spišské Vlachy (J1605) and (b) *St. Ann teaching the Virgin Mary* (J1963) before and after saturation with ethylene glycol (EG); the main visible changes are indicated by arrows; ECM ‐ expandable clay mineral, CH – chlorite group mineral, M ‐ mica group mineral, G – gypsum CaSO_4_.2H_2_O, K – kaolinite Al_2_Si_2_O_5_(OH)_4_, B – boehmite γ‐AlO(OH), Gi – gibbsite Al(OH)_3_

In the analysed micro‐samples, ECMs occurred more often, for example in artworks J1810, J2055, J1605, J1963 or J1843. (Table 4) In order to verify whether ethylene glycol (EG) can interact with the smectite ‐ protein complex (which would allow at least a clear distinction between ECM and chlorites^[21]^), two selected micro‐fragments were exposed directly to EG vapours under conditions identical to those used for standard orientated specimens of clay minerals (6 hours of exposure in 70 °C).^[22]^ In the first case (sample J1605‐4, Figure 3a), there was no significant change in the character and position of a broad diffraction situated in between the micas’ (ca d=1.00 nm) and chlorites’ (d=1.41 nm) 001 diffractions. This weak manifestation of EG saturation can be related to the fact that in this mixed‐layered structure smectite layers are poorly represented (if there are any), and at the same there was apparently a competitive event of EG interaction with gypsum, which is also part of the mixture. The gypsum's most intense diffraction line at d=0.76 nm has disappeared after EG saturation, which could be due to the replacement of water with EG and the formation of an amorphous gel.^[23]^

In the second case (sample J1963‐4B, Figure 3b), there were two diffraction lines next to each other in the initial diffraction pattern ‐ at d=1.41 (probably corresponding to chlorite) and d=1.57 (probably corresponding to smectite or a smectite‐rich interstratified structure affected by proteinaceous binder^[21]^). After saturation with EG vapours, the diffraction at 1.57 disappeared and the intensity of the gypsum line (d=0.67) decreased at the same time. Thus, it can be assumed that in this case the interaction occurred with both the gypsum and the ECM, although the nature of this interaction is not clear. However, it can be considered proven that while the diffraction at 1.41 belongs to chlorite, the diffraction at 1.57 is demonstrably smectite‐rich structure. At the same time, it was confirmed that if a proteinaceous binder is present, smectite 001 line is shifted and can be easily distinguished from the chlorite's one. Due to the ambiguous interpretation of the process of interaction, other samples were not studied. The process of EG saturation in painting layers with ECM affected by the binder requires further systematic research.

### Content of Aluminium

When measuring untreated micro‐fragments of gilding by μXRPD on their surface, it must be taken into account that the layer of the gold/silver preparation (poliment) is very thin, and the composition of the underlying ground layer is always reflected in the result. In all cases, the ground layer was made from chalk ‐ pure calcium carbonate. Therefore, the calcite (CaCO_3_) content was subtracted from the result (Table 4).

**Figure 4:**
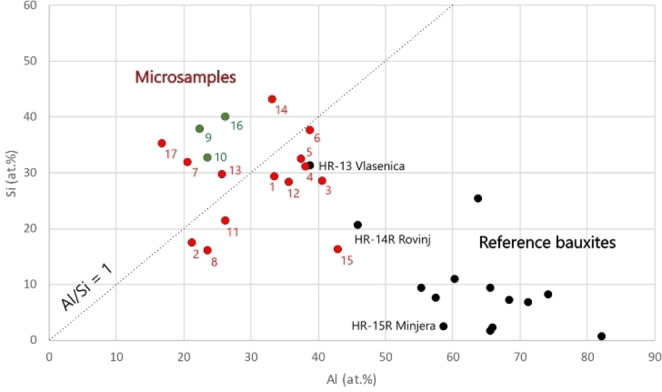
Aluminium and silicon contents in the gilding poliments from the works of art listed in Table 1 (red and green circles) and in the reference bauxite samples (black circles); only artworks represented by green circles contain no Al (oxo)hydroxides or their contents are at the detection limit (less than 1 wt.%); in case of multiple samples from one artwork, values were averaged; 1: J0914‐10, 2: J1324‐9, 3: J1328‐25, 4: J1605‐4, 5: J1606‐6, 6: J1607‐2, 7: J1810‐5,17,21,22 (averaged), 8: J1812‐1,13 (averaged, no μXRPD data available), 9: J1841‐1,2,3,4,6 (averaged), 10: J1843‐6, 11: J1847‐5, 12: J1954‐8,17 (averaged), 13: J1963‐4b, 14: J2012‐7, 15: J2040‐1,2 (averaged), 16: J2055‐3,5 (averaged), 17: J2056‐2

As published in 2017, a positive Al/Si ratio in gilding poliments can be considered as a first indicator of presence of Al (oxo)hydroxides in the layer.^[9]^ However, according to the SEM‐EDS measurements of micro‐samples presented in this paper, it is clear that this criterion is not always met. In Figure 4, artworks containing Al (oxo)hydroxides – gibbsite and/or boehmite and/or diaspore (”bauxite minerals”) are represented by red circles, and only three artworks (marked green) do not contain any of them. These three also have Al/Si ratio less than 1. On the other hand, there are four artworks with Al/Si ratio less than one, yet containing bauxite minerals. In three of them (J2056, J1810, J2055), the content of bauxite minerals is rather small (up to 10 wt.%). Considering the studied period, these three artworks were created quite early (dated to 1470, 1478 and 1503), which could mean that bauxite has been admixed in small quantities already approx. 30 years before the beginning of the 16^th^ century. Consequently, it is possible to imagine that the first bauxite mine or mines could have been mined at a similar time as the alunite mine in Tolfa near Rome (before 1470).

**Figure 5:**
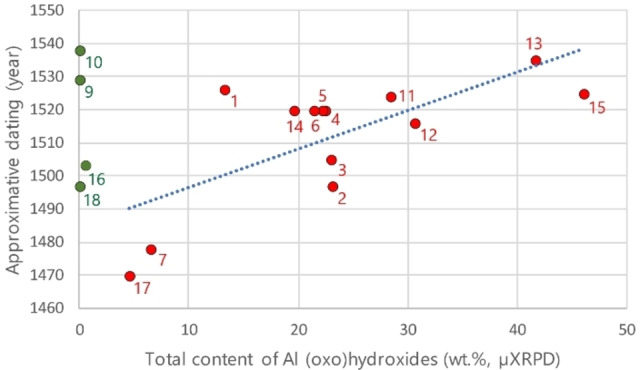
Total content of Al (oxo)hydroxides in relation to the approximate dating of the artwork (colours of circles identical to Figure 4); in case of multiple samples from one artwork, values were averaged; 1: J0914‐10, 2: J1324‐9, 3: J1328‐25, 4: J1605‐4, 5: J1606‐6, 6: J1607‐2, 7: J1810‐5,17,21,22 (averaged), 9: J1841‐1,3 (averaged), 10: J1843‐6, 11: J1847‐5, 12: J1954‐8,17 (averaged), 13: J1963‐4b, 14: J2012‐7, 15: J2040‐1, 16: J2055‐3,4 (averaged), 17: J2056‐2, 18: J2439‐1 (no EDS data available)

Figure 4 shows that the micro‐samples generally contain more Si than the pure bauxite reference samples. This corresponds with their mineralogical composition (Table 4), especially with higher contents of kaolinite, quartz and micas. It may indicate use of clay‐rich bauxites (e. g., from Vlasenica or Rovinj), but unlike kaolinite, neither quartz nor micas are their typical minerals ‐ taking into account the process of bauxite formation in nature.^[24]^ Therefore, it can be assumed that the layer contains a mixture, with bauxite being only one of the components added probably to improve colour or adhesiveness. Therefore, in case of kaolinite, chlorites and ECMs, it is not possible to say with certainty to what extent they were part of the bauxite material and to what extent a part other clay material used in the mixture. Probably the best way to express the amount of bauxite used in gilding preparations is the relative proportion of Al (oxo)hydroxides, because there is no alternative explanation of their presence in painting/gilding. Organic dyes precipitated on an inorganic substrate could be the only alternative material containing aluminium hydroxide, but it is mostly amorphous and in no case can the presence of boehmite or diaspore be assumed in these precipitates. Moreover, no organic dyes were found in the studied poliments. It is very interesting that the amount of Al (oxo)hydroxides seems to increase with artworks created later on, which may be related to the increasing popularity or availability of the new material (Figure 5). Of course, at the same time, there are also works throughout the entire period where bauxite has not been employed in the poliment at all.

### Evidence of Diaspore

Boehmite is the only or significantly predominant Al (oxo)hydroxide in all Istrian bauxites (Rovinj, Minjera, Pazin, Žminj, Šišan), which is consistent with the published data.[^25^, ^26^] It also dominates in the island of Pag and the studied localities in Bosnia and Herzegovina: Vlasenica and Jajce.[^27^, ^28^] Gibbsite is usually one of the significant minerals in bauxites of the North Dalmatian Basin, while the relative amounts of gibbsite and boehmite are quite fluctuating in this area: gibbsite is significantly dominant in the Baraći‐Sinj and Obrovac, where boehmite is only up to 10 wt.%, on the contrary, only 1 wt.% of gibbsite is found in the Drniš and Mostar localities. In Ervenik, the gibbsite:boehmite ratio is close to 1 : 1 and both phases are represented by roughly 30–40 %. (Table 3) These findings are consistent with mineralogical analyses performed at other sites in the area.^[13]^

**Figure 6:**
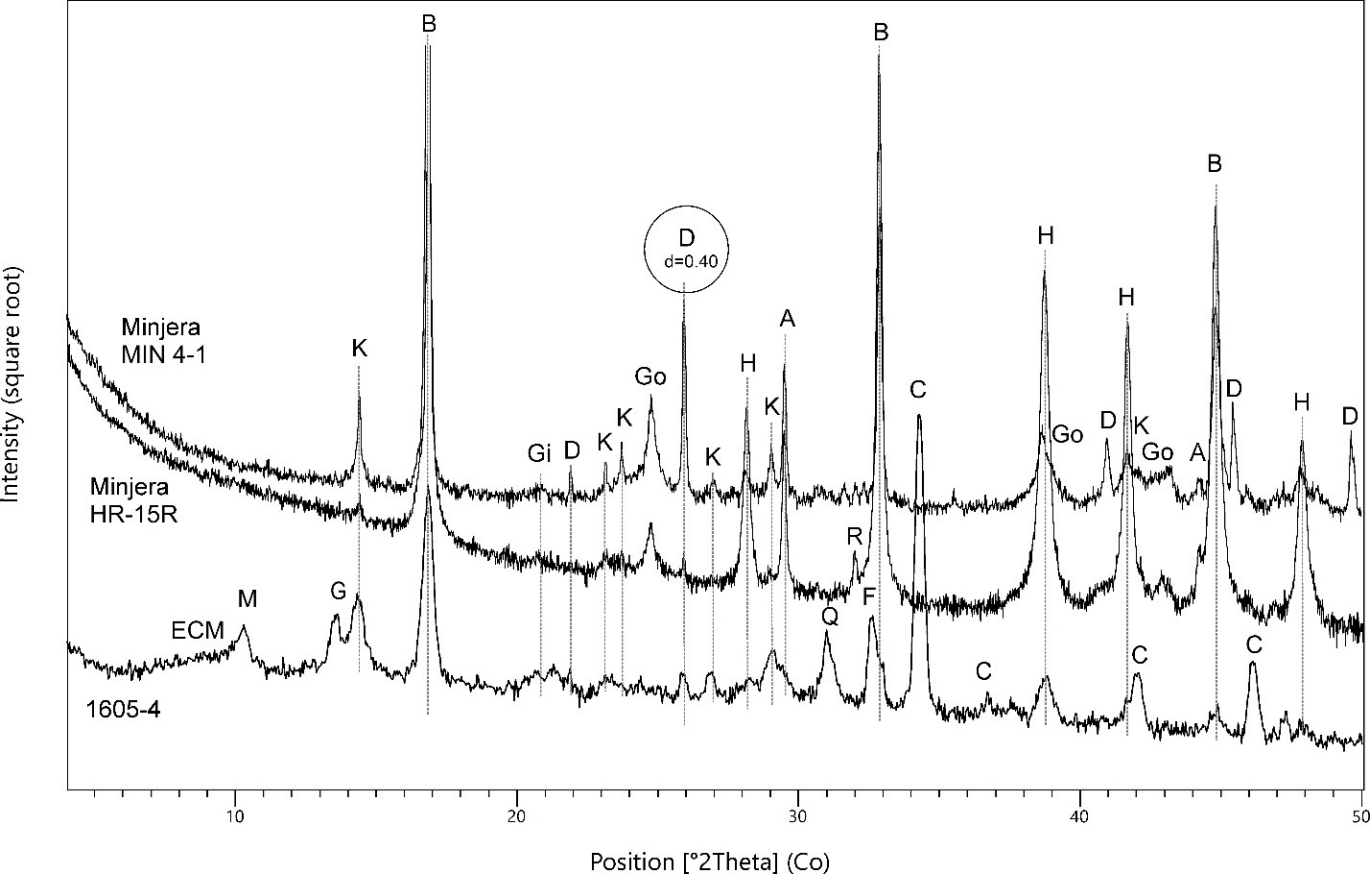
Parts of diffraction patterns of two reference red bauxite samples from Minjera and of the gilding poliment from the polychrome statue of *Crucified Christ* from Spišské Vlachy (J1605, sample 4), showing variable content of diaspore in all measurements; ECM – expandable clay mineral, M ‐ mica group mineral, G – gypsum CaSO_4_.2H_2_O, K – kaolinite Al_2_Si_2_O_5_(OH)_4_, B – boehmite γ‐AlO(OH), Gi – gibbsite Al(OH)_3_, D – diaspore α‐ AlO(OH), Go – goethite α‐FeO(OH), H – hematite Fe_2_O_3_, A – anatase TiO_2_, R – rutile TiO_2_, Q – SiO_2_, F – feldspar/plagioclase – (Na,Ca)AlSi_3_O_8_, C – calcite CaCO_3_; the most intense diffraction of diaspore correspond to interplanar distance d(110)=0.4 nm.

The most interesting is probably the variable and sometimes very high content of diaspore in the Minjera locality, which is consistent with the previously reported data.[^8^, ^11^, ^29^] Diaspore was not found in any other reference sample in this study. However, in a much smaller quantity, it has been previously documented in Rovinj grey bauxites, because its formation is naturally associated with pyritization and resilicification (formation of kaolinite) during bauxite diagenesis.^[8]^ Therefore, its other, although not yet published, occurrences cannot be ruled out wherever bauxite pyritization has occurred. Pyritized bauxites usually form irregular bodies inside red and yellow‐red bauxites, and besides Minjera and Rovinj, pyritization has been described in other Istrian locations ‐ Grdo Selo near Pazin, and Blatna Ves near Roč.^[7]^ In the studied works of art, diaspore was uniquely proven as a minor phase in two cases – on the wooden sculptures of the *Death of the Virgin Mary* dated to 1478 (J1810) and *Crucified Christ* from around 1520 (J1605, Figure 6), both located in the Spiš region, Slovakia. At least in these two cases, where diaspore is found, Minjera seems to be a very likely source location for bauxite. Although gibbsite is not described in the literature at any Istrian locality, it was newly identified in a sample of grey bauxite from Minjera HR‐15G (Table 3). In this context, it is therefore interesting that the combination of dominant boehmite, and minor diaspore and gibbsite occurs in the gilding poliment of the *Crucified Christ* from around 1520 (micro‐sample J1605) – similarly to Minjera.

Regardless of the great variability of the mineralogical composition within one locality (e. g., Minjera, Table 3), it is certain that gibbsite is not the dominant bauxite mineral anywhere in Istria. Nevertheless, part of the investigated poliments (previously published and also newly measured) mainly contains gibbsite. Therefore, it seems clear that the source localities must have been at least two, not just Minjera.

**Figure 7:**
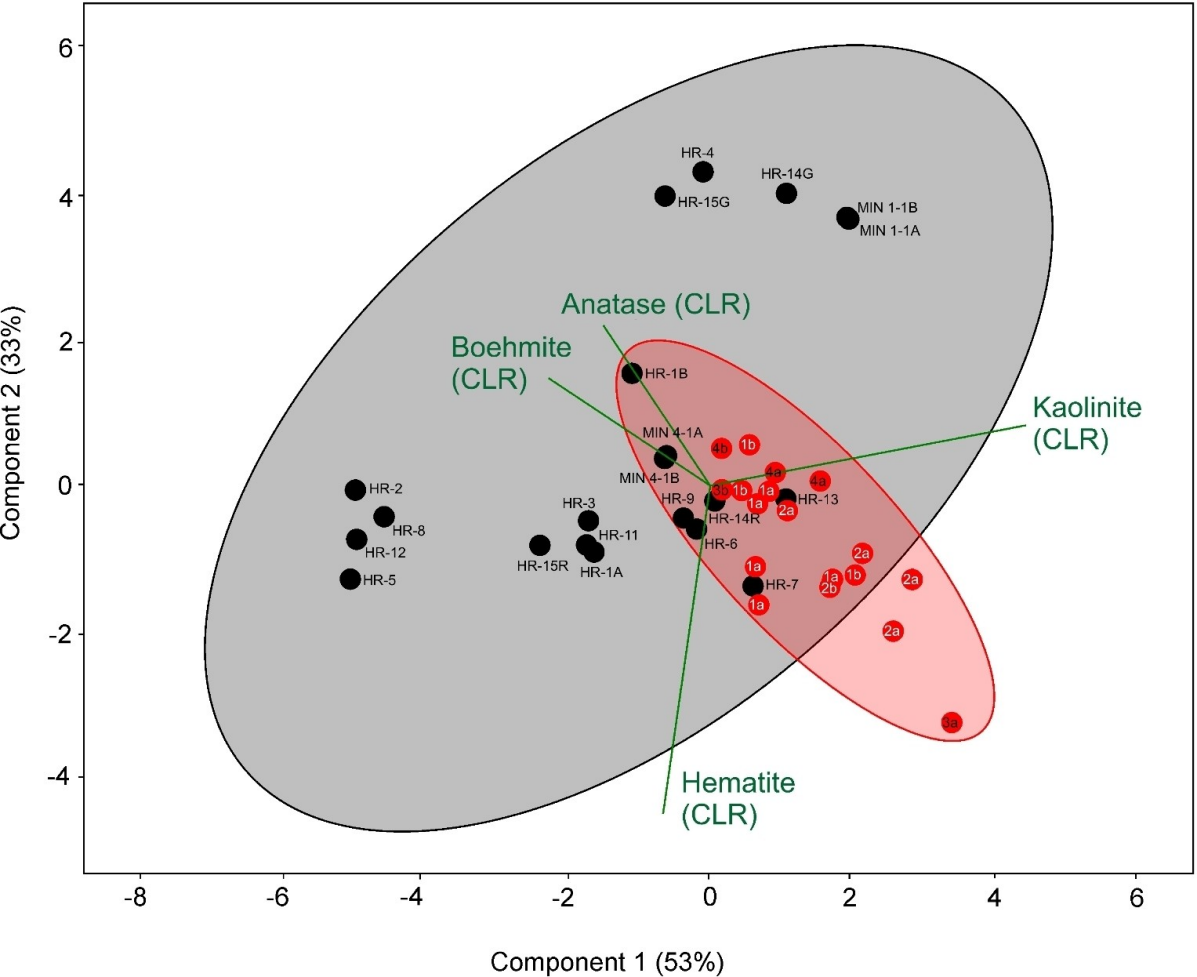
Compositional biplot of CLR‐transformed variables^[32]^ depicting variation of main bauxite components – boehmite, kaolinite, hematite and anatase in reference samples (black circles) and poliments (red circles); bauxites with the most similar ratio of the main phases are found in the intersection of the respective sets; CLR=centre log ratio (for details see Experimental Section), HR−X or MIN X=reference bauxite codes (see Table 1); numbers and letters in red circles: 1 – group of artworks belonging to the workshop of Master Paul of Levoča, Slovakia (J1954, J1605, J1606, J1607, J2040, J0914, see Table 2), 2 – other artworks from the Spiš region, Slovakia (J6502, J1810, J2055, see Table 2), 3 – artworks located in the Ore Mountains region at the Czech‐Saxon border, Czechia (J2012, J1847, see Table 2), 4 – artworks located in other regions of the Czech Republic, created by German masters (J1324, J1328, J1963, see Table 2); a – polychrome wooden sculptures, b – panel paintings; one artwork may be represented by multiple samples; samples and artworks containing no bauxite minerals (Table 4) are not included.

In order to further specify the place of extraction of the raw material used in the 15^th^ to the 16^th^ century, variation of the minerals regularly found in karst bauxites (boehmite, kaolinite, hematite and anatase) was plotted (Figure 7). A relatively high concentration of these minerals in bauxites prevents significant ratio change if some of these minerals were preferably enriched during their processing for gilding purposes, and/or if they were added in small quantities with other components (quartz, mica etc.). It was assumed that this contamination is insignificant since there is no positive correlation between bauxite minerals and those which are fully added (quartz, mica).

As compositional biplot suggest (Figure 7) variation of bauxite minerals found in poliments is very similar to some raw bauxite samples. More precisely, it is a reasonable assumption to consider samples from Minjera (MIN 4–1), Rovinj (HR‐14R), Vlasenica (HR‐13) and the North Dalmatian Basin (Baraći‐Sinj – HR‐7, Obrovac – HR‐9) and Islands (Pag – HR‐6) as the most suitable candidates to be possible materials for the poliment production in the 16^th^ century. However, there is no suitable reference locality in the selection for dominantly gibbsite‐rich poliments. It is also clear that the colour (orange‐red) was important for the choice of material for the poliments and, therefore, the darker hematite‐rich samples, including the second sample from Minjera (MIN‐15R), fall outside. Of course, the grey samples do not fit either.

In this context, it is interesting that three poliments on three different pieces belonging to the same workshop (Master Paul of Levoča) have different compositions. The poliment on both micro‐fragments taken from the wooden sculpture of St. Ann from the main St. James’ Church in Levoča, Slovakia (J2040), contains just boehmite and at the same time it is the poliment with the highest bauxite minerals’ content among the studied samples. The poliment on the other altar (St. George, J1954) from the same church (Church of St. James, Levoča), however, contains predominantly gibbsite in all collected micro‐samples, with only a small proportion of boehmite. and finally, wooden sculpture of *Crucified Christ* from Spišské Vlachy, Slovakia (J1605) contains, in addition to boehmite, gibbsite and especially diaspore, indicating Minjera. It means that one workshop bought poliments from different sources or suppliers, or the mineralogical composition within one product must have had to be quite variable on the past.

### Gypsum and Technology of Gilding

Gypsum has not been identified in any reference sample (except for a very small amount detected in grey bauxite from Minjera, Table 2). In gilding poliments, however, it is found as an admixture in all studied cases, with the exception of one panel painting (J1834) and one sculpture (J1841), which both do not contain any bauxite minerals. The gypsum admixture usually varies in units of % and it seems likely that gypsum was deliberately mixed into the material.

According to mediaeval recipes for “water gilding”, the gold leaf to be burnished was laid either directly onto the moistened ground (chalk‐ or gypsum‐based) or over an intermediate layer of a moistened reddish poliment, which enriched the colour. Originally, the poliment contained fine‐grained red clay, the so‐called bole. It is expressly stated, that without the support of a gypsum or a bole, the gold would tear.^[30]^ If bauxite was used instead of bole, even mixed with some other clay, the addition of gypsum may have been necessary to allow the gold to be polished. However, the source of gypsum remains a question, as some researchers not believed to have been available in the regions north of the Alps during the Gothic and Renaissance periods.^[31]^

**Figure 8:**
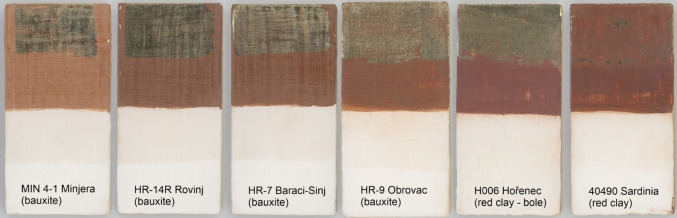
Technological replicas – various bauxites and red clays applied in thin layers on wooden plates primed by white chalk and polished before application of a gold foil on the top (photo: J. Hradilová, full explanation in the text)

Technological experiments were performed to evaluate the suitability of different bauxites for water gilding applications. To reflect the contemporary practice, gypsum was admixed to bauxite, albeit in a very small amount. As it is not possible to quantitatively determine the ratio of inorganic and organic components in the micro‐fragments of poliments, it was decided to use a rather lower proportion of the glue binder (glue:bauxite=1.5 : 1) in order to highlight the differences between the individual bauxites; no clay was admixed. Bauxites with the closest composition to the studied poliments (Figure 7), i. e. from Minjera (MIN 4–1, boehmite‐diaspore type), Rovinj (HR‐14R, boehmite type), Baraći‐Sinj (HR‐7, gibbsite type) and Obrovac (HR‐9, gibbsite‐boehmite type) were selected for comparison. Another considered factor was the suitable colour and content of the clay component, varying from 34 wt.% in Rovinj (clayey bauxite) through 17 wt.% (Baraći‐Sinj) and 14 wt.% (Minjera) to only 8 % (Obrovac). The sample from Vlasenica (HR‐13) was not used, although its composition is satisfactory. The historical context shows that, unlike the coastal and more western locations in Dalmatia, this territory had been occupied by the Turks already in the second half of the 15^th^ century, and access to this source for the Saxons was, therefore, prohibited. Compared to the two red clays (kaolinitic bole H111 and kaolinit‐smectitic clay 40490) available today, which were technologically processed in the same way only without the addition of gypsum, the selected bauxites performed surprisingly well. The resulting gildings are depicted in Figure 8. The bauxites were assessed in the following categories: workability, polish ability, adhesive power and final colour.

*Workability*. Fine fraction was obtained by dissociation in distilled water. Workability was mainly influenced by the degree of rock solidification, as the reference bauxite samples did not contain any coarse‐grained minerals. It was also found by XRPD that the fine fraction and the whole rock composition are identical. The most difficult to process were the compact samples of gibbsite‐rich bauxites from the Baraći‐Sinj and Obrovac localities, Minjera samples were processed without troubles, and the soft clayey sample from Rovinj was the best. The reference clays were not processed because they were obtained in the ready‐to‐use (powdered) form.

*Polish ability*. The bauxite from Minjera was the best polished (completely comparable to both red clays). On the other hand, the samples from Baraći‐Sinj and Obrovac were the hardest to polish. Bauxite from Rovinj was average from this point of view.

*Adhesive power*. Contrary to expectations, the red clays behaved quite differently ‐ while the kaolinitic Czech bole held the gold perfectly, in the case of the commercially available kaolinitic‐smectitic Italian clay, the result was the worst and the gold fell off. Bauxites from Rovinj and Minjera also had perfect adhesiveness fully comparable to the Czech bole. Bauxites from Baraći‐Sinj and Obrovac were worse, but the result was still acceptable.

*Colour*. It is not easy to evaluate the colour, as it is very variable even within each locality. Regarding the selected samples, the lightest shade was represented by Minjera, the other bauxites were darker, having a similar colour to the employed red clays or slightly brownish. All the samples are within a colour range possible from the point of view of historical technology.

## Conclusions

It was proved that bauxite had been used in Central European preparation layers under gilding/silvering (poliments) at least in the period 1470–1550. Its extent is variable, as it had been an ingredient improving the properties of apparently lower‐quality clay raw materials with higher proportion of quartz. The discovery of boehmite in artwork dated to 1470 is apparently the evidence of earliest intentional use of red bauxite in technology.

As the investigated artworks are related to prosperous mining regions, a connection with the activities of Saxon prospectors and miners is likely. The use of red bauxite in poliments could be related to the mining of grey bauxite for the production of alum in Istria, Croatia (Minjera site). This possibility is supported by the finding of diaspore in two studied artworks. It is so far the only demonstration of the presence of diaspore in painting.

Variable abundance of boehmite and/or gibbsite could indicate several different sources, not only Minjera, as follows from the comparison with reference samples from Istria and the Balkan Peninsula. Unfortunately, it is not possible to use clay minerals to determine the provenance more precisely, because the materials probably contain a mixture from several sources, and in addition, a closer identification of expandable clay structures is complicated by the interaction with organic binder in the paint.

Technological tests have proven the suitability of bauxite as a preparation for gold plating. It is thus obvious that the cheaper bauxites at the turn of the 15^th^ and the 16^th^ centuries could actually replace the red clays (the so‐called boles) imported for this purpose from the Mediterranean. The search for alternative sources in this period is related to the interruption of trade routes due to Turkish invasion. Bauxite could thus replace both imported alum and also the so‐called Armenian bole (= red clay for goldsmiths and painters).

These new findings fundamentally change the long‐held ideas and misconceptions that the preparations for water (poliment) gilding of panel paintings, sculptures and carvings was always based on high‐quality clay – the bole. Other alternative materials were never considered. It now seems likely that bauxites replaced imported boles in the transitional period of the early modern era, before the mining of local coloured clays (earths) began in Europe and before they became widespread in the Baroque period. The relatively narrow range of bauxite use in poliments can potentially also serve for the purposes of relative dating of anonymous works of art.

## Experimental Section

### Light Microscopy

An Axio Imager A2 microscope (Zeiss, Germany) was used to observe cross‐sections in reflected white light in combination with side illumination and UV (Colibri 2 system with 365 nm UV and 470 nm LED module); photographs were taken with an Olympus DP74 digital camera with CS‐ST + EFI module.

### Scanning Electron Microscopy/Microanalysis (SEM‐EDS)

All cross‐sections were also analysed using a Jeol JSM6510 scanning electron microscope equipped with an energy dispersive spectrometer INCA (Oxford Instruments) with a silicon drift detector allowing detection of elements heavier than Be (Z >4) at 125 eV resolution. Measurements were carried out in low‐vacuum mode under 50 Pa pressure and 20 kV accelerating voltage; backscattered electrons were detected. A low‐vacuum mode allowed analysis of the samples without conductive coating of their surface. Standardless quantification using ZAF correction (Genesis Spectrum SEM Quant ZAF, version 3.60) was applied to calculate the elemental composition.

### X‐ray Powder Diffraction (XRPD)/Micro‐diffraction (μXRPD)

Diffraction patterns of the reference powdered samples were collected with Malvern PANalytical Empyrean, Series 3 diffractometer equipped with a conventional X‐ray tube (Co Kα radiation, 40 kV, 30 mA, line focus), multicore optics, and a linear position sensitive detector PIXCel3D detector. Conventional Bragg‐Brentano geometry was used. X‐ray patterns were collected in the range of 4°–80° 2θ with a step of 0.013° and 600 s/step produces a scan of about 3 h and 56 min. Samples were filled into sample holders with the back loading technique. X‐ray diffraction patterns were not pre‐treated before interpretation as no background correction was needed. Qualitative analysis was performed with the HighScore Plus software package (Malvern PANalytical, version 5.2.0)^[33]^ and PDF‐5+ database.^[34]^

Untreated micro‐fragments were subjected to μXRPD analysis. Diffraction patterns were collected with a PANalytical X'PertPRO MPD diffractometer equipped with a conventional X‐ray tube (Co Kα radiation, 1.7890 Å, 40 kV, 30 mA, point focus). A glass collimating mono‐capillary with exit diameter 0.1 mm was used in the primary beam. A multichannel position‐sensitive detector X'Celerator with an anti‐scatter shield and Fe beta filter was used in the diffracted beam. X‐ray patterns were taken between 4 and 80° 2θ with 0.033° step and 2200 s counting time per step, producing a total counting time of about 12 h. Qualitative phase analysis was performed using the HighScorePlus software package (version 5.2.0, Malvern PANalytical, Netherlands)^[33]^ and current PDF‐5+ database.^[34]^

Clay minerals were interpreted according to Moore and Reynolds.^[22]^ Quantitative phase analysis (QPA) was performed using the Rietveld method.^[35]^ As the clay minerals exhibit a wide range of disorders (stacking faults in the layer structure), the Profex 5.4.0/BGMN 4.2.23 software was used for all calculations.[^36^, ^37^] This program includes a code which permits the use of structural models correctly describing the disorder models.[^38^, ^39^, ^40^] Structural models of other phases were described as standard Rietveld models.^[41]^ The main limitations for the use of quantitative analysis for micro‐samples of paintings are described by, e. g., Švarcová *et al*.^[42]^ Despite these limitations, QPA based on μXRPD data provides a reliable basis for estimation of phase quantities in micro‐samples.

### Computational Methods

The contents of four main bauxite minerals (boehmite, kaolinite, hematite and anatase) were selected for statistical processing. Since quantitative mineralogical data are compositional by their nature^[10]^ and all information is stored in ratios between components, a conventional statistical approach was not suitable. Sample space of compositional data is called simplex (*S* ^*D*^), with the following definition:

$$s^{D}=\left\{x=\left.\left[x_{1},x_{2,\cdots }x_{D}\right]\right|x_{1}>0,\;i=1,\;2,\cdots D;\sum_{i=1}^{D}x_{i}=K\right\}$$

where K is a constant (usually 1 or 100). It is clearly visible from the definition that zeros are not allowed in the dataset. There are several strategies how to overcome this difficulty regarding the nature of the zero.[^43^, ^44^] In this case, zeros were replaced by a very small number (0.1), since in the case of the main bauxite components, zero may also stand for low concentration under detection limit and not the true absence. In the second step, the data were transferred from the simplex into regular Euclidean space by centre log ratio (CLR) transformation defined as:

$$clr\left(x\right)=\;\left[ln\frac{x_{1}}{g_{m}\left(x\right)},\;ln\frac{x_{2}}{g_{m}\left(x\right)},\cdots ,ln\frac{x_{D}}{g_{m}\left(x\right)}\right],$$

where g_m_(x) is the geometric mean of the components in *x*. Variation of the CLR‐transformed variables was analysed by the compositional biplot,^[32]^ which has a different interpretation compared to the conventional principal component analyses (PCA).^[43]^ Analyses were performed in CoDa pack 3d.^[45]^ Since ratios between minerals, which are characteristic of a source, are presumably preserved from source to the poliment (final product) in this way a link between source and poliment can be established.

### Experimental Gilding

The washed out fine portion of the bauxite or clay was mixed with an aqueous 7 % solution of animal glue in a ratio of 1 : 1.5 (1.33 g of bauxite/clay to 2 g of glue), rubbed in a bowl, then strained 3 times through a sieve to make the consistency as fine as possible. Subsequently, 4 layers of the mixture were applied to an oak plate previously provided with a thin layer of white chalk ground and proteinaceous insulation. After 4 hours, the surface was lightly smoothed with fine emery (1200), lightly polished with agate and deerskin. On the second day, one layer of gold foil was applied with the help of 40 % ethanol. After approx. 3–4 hours, the gold was lightly polished again with agate and deerskin.^[46]^

## Conflict of Interests

The authors declare no conflict of interest.

## Data Availability

The data that support the findings of this study are available from the corresponding author upon reasonable request.
